# DNA polymerase γ and disease: what we have learned from yeast

**DOI:** 10.3389/fgene.2015.00106

**Published:** 2015-03-17

**Authors:** Tiziana Lodi, Cristina Dallabona, Cecilia Nolli, Paola Goffrini, Claudia Donnini, Enrico Baruffini

**Affiliations:** Department of Life Sciences, University of ParmaParma, Italy

**Keywords:** DNA polymerase γ, Mip1, Mip1 interactions, Pol γ mutations, yeast model

## Abstract

Mip1 is the *Saccharomyces cerevisiae* DNA polymerase γ (Pol γ), which is responsible for the replication of mitochondrial DNA (mtDNA). It belongs to the family A of the DNA polymerases and it is orthologs to human POLGA. In humans, mutations in *POLG(1)* cause many mitochondrial pathologies, such as progressive external ophthalmoplegia (PEO), Alpers' syndrome, and ataxia-neuropathy syndrome, all of which present instability of mtDNA, which results in impaired mitochondrial function in several tissues with variable degrees of severity. In this review, we summarize the genetic and biochemical knowledge published on yeast mitochondrial DNA polymerase from 1989, when the *MIP1* gene was first cloned, up until now. The role of yeast is particularly emphasized in (i) validating the pathological mutations found in human *POLG* and modeled in *MIP1*, (ii) determining the molecular defects caused by these mutations and (iii) finding the correlation between mutations/polymorphisms in POLGA and mtDNA toxicity induced by specific drugs. We also describe recent findings regarding the discovery of molecules able to rescue the phenotypic defects caused by pathological mutations in Mip1, and the construction of a model system in which the human Pol γ holoenzyme is expressed in yeast and complements the loss of Mip1.

## Introduction

DNA polymerase γ (or Pol γ) is the only DNA replicase identified in animal and fungal mitochondria. Although four other DNA polymerase activities were recently identified in *Saccharomyces cerevisiae* mitochondria (DNA polymerase ζ, Rev1, DNA polymerase η, and DNA polymerase α) (Zhang et al., 2006; Chatterjee et al., 2013; Lasserre et al., 2013), Mip1, the yeast Pol γ, is the only enzyme to be able to fully replicate mitochondrial DNA (mtDNA). Deletion of *MIP1* makes the strain *rho*^0^, i.e., devoid of mtDNA.

*S. cerevisiae* is a facultative anaerobe yeast and is able to survive in the absence of oxidative phosphorylation (OXPHOS) activity and of mitochondrial genome, provided that a fermentative carbon source is available. The non-essentiality of respiration for viability makes this organism an excellent model for studying mitochondrial biogenesis as well as nucleo-mitochondrial interactions. Respiratory deficient (RD) cells efficiently generate the ATP needed to sustain the growth by alcoholic fermentation. On media containing a limiting amount of fermentative carbon sources, colonies generated by RD cells display the so called “*petite* phenotype”, since they are smaller than the respiratory proficient wild type colonies because they are unable to produce biomass through respiration of ethanol, the end product of fermentation. Since both nuclear and mitochondrial genomes participate to OXPHOS functions, the *petite* phenotype can derive from mutations in nuclear genes that encode mitochondrial functions (*pet* mutants, Mendelian inherited, Sherman, 1963) or from mtDNA mutations (*petite* mutants, non-Mendelian inherited, Ephrussi and Slonimski, 1955). Cytoplasmic *petite* mutants, which arise spontaneously with high frequency (around 1–10% depending on the strains), are completely devoid of mtDNA (*rho*^0^ cells) or carry extensive deletions of mtDNA and regular repetitions of the conserved sequences (*rho*^−^ cells). Cells containing functional mitochondrial genomes are termed *rho*^+^ (Dujon, 1981). *S. cerevisiae* is sensitive to antibiotics that inhibit the mitochondrial translation, such as erythromycin, when grown on oxidative carbon sources. Erythromycin resistant mutants arise spontaneously with low frequency (10^−7^ to 10^−8^) as a result of mutations in specific nucleotides of the mitochondrial gene encoding the 21S rRNA (Sor and Fukuhara, 1984). For this reason, in yeast strains mutated in *MIP1* the frequency of mutants resistant to erythromycin (Ery^R^ mutants) is an *in vivo* index of the replication fidelity of Mip1.

Each yeast cell contains 10–50 copies of mtDNA per nuclear genome, depending on the growth condition. Recombination between these molecules is highly active (Dujon et al., 1974). A strong link between erroneous recombination and *rho*^−^ transmission has been postulated, which would explain the molecular mechanism that generates this class of cytoplasmic *petite*. In particular, it has been suggested that mtDNA deletions giving rise to *rho*^−^ genomes can occur through homologs recombination between imperfect repeats (Slonimski and Lazowska, 1977; Gaillard et al., 1980). *Rho*^−^ mtDNA genomes are not very stable and may then evolve into *rho*^0^ status. *Rho*^0^ clones can also be generated directly by treatment with different molecules, such as ethidium bromide (EtBr), which inhibits both mitochondrial transcription and mtDNA replication, either directly or indirectly (Slonimski et al., 1968; Richardson and Parker, 1973). Human tissues, in case of mtDNA mutations, are mostly heteroplasmic, whereas yeast is homoplasmic: if mtDNA point mutations or mtDNA rearrangements occur, after a few generations two populations of cells will be produced, one bearing only wild type mtDNA molecules and the other bearing only mutated mtDNA molecules.

mtDNA is packaged within protein-DNA structures called nucleoids, which are anchored to the inner mitochondrial membranes (Chen et al., 2005). A variable number of nucleoids are present in each mitochondrion and each nucleoid contains several copies of mtDNA. Different proteins make up nucleoid structures, and are all involved in maintaining the integrity of the mtDNA and are responsible for its replication, recombination, repair and transmission to the bud (Chen and Butow, 2005; Kucej et al., 2008). Mutants which have been altered or are devoid of these functions are heavily affected with respect to mtDNA stability. Therefore, depending on the nuclear background, the level of cytoplasmic *petite* mutants can increase, in some cases reaching the entire cell population.

The mechanism of mtDNA replication in yeast is not completely known. In contrast to what has been assumed for decades, mtDNA is not always circular, as in the case of animals. The majority of mtDNA is present as concatenamers of linear tandem arrays of several genome units and only a small proportion of mtDNA has a circular shape (Maleszka et al., 1991). In particular, concatenamers are mainly present in mother cells whereas circular mtDNA is found in the buds (Ling and Shibata, 2002). Two theories were proposed regarding the replication of yeast mitochondrial DNA. (i) According to the most accepted theory, the replication starts at several origins called Ori sites, and it is RNA-primed and bidirectional like that of chromosomal DNA (reviewed in Lecrenier and Foury, 2000). While Ori sites have not been identified in other fungi, it is known that Ori sites of *S. cerevisiae* are 300-bp-long sequences composed of three repeated GC-rich clusters separated by an AT-reach region and preceded by a transcription site called r (Baldacci et al., 1984; Foury et al., 1998). (ii) According to the second hypothesis, the replication occurs via a “rolling circle” mechanism, which produces long tandemly repeated mtDNA molecules, which are then converted into circular monomers (Ling and Shibata, 2002; Ling et al., 2007). Recent findings also demonstrate that homologs recombination and strand invasion could account for initiating replication in yeasts (Ling and Shibata, 2002; Ling et al., 2007). Therefore, the mtDNA replication mechanism in yeast is different from the best-known mechanism in mammalians, in which both the H and the L strands are continuously synthesized from OriH and OriL, respectively, which are located far from each other. In this case, two models have been proposed. (i) According to the most accepted hypothesis, i.e., the asynchronous strand displacement model, at the beginning the H strand is replicated by single-stranded replication starting from the OriH, with displacement of the D-loop. This synthesis proceeds until OriL is exposed. In the OriL site, synthesis of the L-strand is initiated in the opposite direction and is primed by RNA synthesis (Shadel and Clayton, 1997). (ii) Alternatively, the strand-coupled bidirectional replication model has been proposed, in which bidirectional replication is initiated from a region near OriH, followed by progression of the two forks around the mtDNA circle (Holt et al., 2000). More recently other mechanisms have been proposed (reviewed in McKinney and Oliveira, 2013). A limited number of proteins are involved in mtDNA replication, and most of them are conserved in yeast and humans, which suggests that the molecular mechanisms of the replication are partially similar (reviewed in Lecrenier and Foury, 2000).

## Mip1 milestones: a historical point of view

The first information on yeast DNA polymerase γ dates back to 1970, when a mitochondrial DNA polymerase was proven to be resistant to aphidicolin and highly sensitive to ddNTPs, such as DNA polymerase γ of higher eukaryotes (Wintersberger and Wintersberger, 1970; Wintersberger and Blutsch, 1976). The *MIP1* gene was cloned by functional complementation of a thermosensitive (ts) mutant, called *mip1-1*, identified in a screening of mutants able to grow on glycerol at 25°C but not at 36°C, due to massive production of *petite* mutants at high temperatures (Genga et al., 1986). In particular, in these conditions the *mip1-1* mutant was completely deficient of both mtDNA replication and mtDNA polymerase activity (Foury, 1989). The gene was mapped to chromosome 15 and sequenced, resulting in an open reading frame encoding a 1254 amino acid long protein. The deletion of *MIP1*, as expected, produced a strain that was unable to grow on respiratory carbon sources and devoid of mtDNA. Cloning *MIP1* paved the way for the isolation of genes encoding the DNA polymerase γ of *Schizosaccharomyces pombe* (Ropp and Copeland, 1995) and, subsequently, of humans and *Drosophila* (Ropp and Copeland, 1996).

Based on the alignment with bacterial DNA polymerases of family A, which includes bacterial and bacteriophage polymerases which share significant similarity to *E. coli* polymerase I, three highly conserved motifs were identified in the exonuclease domain and called *exo1*, *exo2*, and *exo3* (Bernad et al., 1989). A mutation in *exo1*, a mutation in *exo2* and two single mutations in *exo3* were introduced in Mip1 (Foury and Vanderstraeten, 1992). These mutations determined a decrease in proofreading activity and consequently a mtDNA mutator phenotype, characterized by an increased frequency of Ery^R^ mutants, which demonstrates the functional role of the *exo* motifs. However, in these mutants an increase of mtDNA extended mutability, accompanied by a decrease in the gap-filling activity and processivity, was also observed, especially at 35°C. This suggests that the two catalytic domains do not act independently of each other in Pol γ. The discovery that the D230A mutant displayed a significant reduction of exonuclease activity was the basis for the creation of a mouse model lacking Pol γ exonuclease activity. In this model, an increase in mtDNA point mutability and deletions, a reduction in the life span and the onset of premature aging were observed (Trifunovic et al., 2004).

**Figure 1:**
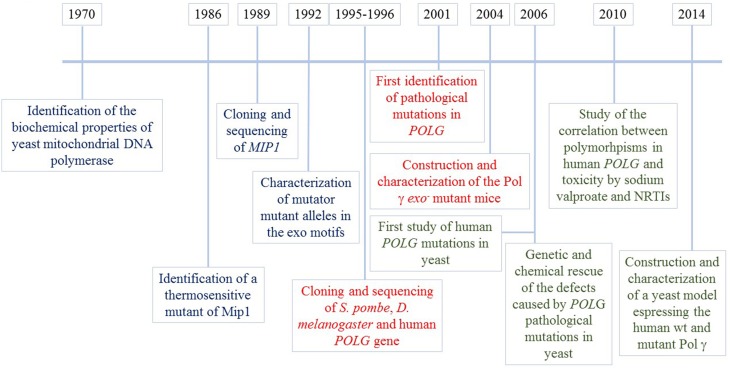
**Mip1 milestones**. Information regarding yeast Mip1 is shown in blue, information on other eukaryotic Pol γ obtained thanks to the use of yeast Mip1 is in red, information on human POLGA mutations/polymorphisms obtained by modeling and studying the mutations in yeast is in green.

Thanks to its ability to grow even in the absence of mitochondrial DNA and to the sequence conservation among eukaryotic polymerase γ, yeast was considered to be the organism of choice to study the effects of pathological mutations in human Pol γ, starting from 2006 (Stuart et al., 2006), a few years after the first identification of pathological mutation in the *POLG* gene (Van Goethem et al., 2001) Several works have been published since then, in which *mip1* alleles carrying substitutions corresponding to pathological mutations were expressed in mutant strains devoid of mtDNA polymerase, as explained later. Besides studies designed to validate the pathogenicity of human mutations and to understand the molecular mechanisms responsible for the associated diseases, studies performed in yeast led to the discovery of the genetic and chemical rescue of the effects of mutations in *MIP1*, through ribonucleotide reductase overexpression and the administration of antioxidants, respectively (Baruffini et al., 2006). These observations led to several studies on human cells or murine organisms. At the same time, with the help of yeast, pharmacogenetic research was performed in order to study the correlation between toxicity due to sodium valproate or NRTIs, and polymorphisms in *POLG* (Baruffini and Lodi, 2010; Stewart et al., 2010). The milestones described in this chapter are illustrated in Figure 1.

## Mip1 biochemical properties

**Figure 2:**
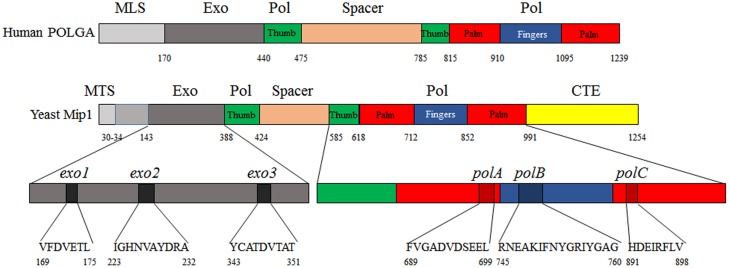
**Domains of human POLGA and yeast Mip1 shown in linear form**. Linear form of human POLGA according to its 3D structure. The linear form of Mip1 was constructed following the alignment of yeast Mip1 and human POLGA. MLS, mitochondrial localization signal; Exo, exonuclease domain; Spacer, spacer or linker region; Pol, polymerase domain; MTS, mitochondrial targeting sequence; CTE, C-terminal extension.

Mip1 is a protein of approximately 140 kDa, it belongs to the subclass γ of the family A polymerases to which several bacterial and viral DNA polymerases belong, and is divided into four domains: besides a mitochondrial targeting signal (MTS) motif, which is necessary for the import into the mitochondrial matrix, it possesses an exonucleasic (exo) domain, a spacer or linker region, a polymerase (Pol) domain and a C-terminal extension (CTE) specific to fungi polymerase γ (Figure 2).

Thanks to the purification of the native protein from *S. cerevisiae* (Sen et al., 1994), it has been possible to determine the kinetic properties of Mip1 polymerase. Mip1 has a K_*m*_ for dNTPs lower than 1 μM (Sen et al., 1994), while the k_pol_ is about 60 nt/s and the processivity is about 500 nt per one binding event (Viikov et al., 2011). It can use different types of DNA as substrates, including the poly [dA-dT] and poly[dA]-oligo[dT], but not the poly [rA]-oligo [dT]. The inability to use this product as a substrate distinguished Mip1 from Pol γ of higher eukaryotes. The following molecules act as inhibitors of the catalytic activity: the Mn^2+^ ion, whose *in vivo* administration increases up to 100 times the mtDNA point mutability (Putrament et al., 1975), EtBr, ddTTP, and ddCTP as well as various nucleoside reverse transcriptase inhibitors (NRTIs) which are also used in antiretroviral therapy, such as 3′-fluoro-TTP and didehydro(d4)CTP (Sen et al., 1994; Eriksson et al., 1995). Furthermore, Mip1, similarly to prokaryotic polymerases of family A, is also able to perform strand displacements with rates of about 30 nt/s (Viikov et al., 2011), in accordance with the observation that human Pol γ can be stimulated to perform strand displacement by the human DNA2 protein (reviewed in Copeland and Longley, 2008).

The polymerase domain is divided into three subdomains: thumb, palm and fingers. Three motifs, which are highly conserved in all eukaryotic Pol γ, are located in the Pol domain and are called *polA*, *polB*, and *polC*. The first mutants in the catalytic domain were constructed by random mutagenesis in 1995 (Hu et al., 1995). Three mutations in the fingers subdomain, T716I, E724K, and P851L, were associated both with increased point mutability and increased extended mutability (Table 1), indicating that the catalytic domain is involved both in the mtDNA polymerization and in replication fidelity. Moreover, these mutations are partially dominant, as are most of the pathological substitutions located in the fingers subdomain identified in patients with adPEO.

**Table 1:** **Substitutions which alter *in vivo* mtDNA mutability and/or *in vitro* biochemical properties**.

**Substitution**	**Domain**	***Petite* frequency^a^**	**Increase in Ery^R^ frequency**	**Thermosensitivity^b^**	**Dominance regarding *petite* frequency**	**Polymerase activity^c^**	**Processivity^c^**	**Exonuclease activity^c^**	**References**
D171G	Exo	3.9–4.1%	≅100–180	Moderate			↓	↓↓	Foury and Vanderstraeten, 1992; Vanderstraeten et al., 1998
D171G/D230A	Exo/Exo	31.5%	≅1440	Very strong			↓↓	↓↓	Vanderstraeten et al., 1998
E173K	Exo	As wt	≅110–210	Strong	Recessive				Hu et al., 1995; Vanderstraeten et al., 1998
S198L	Exo	23%	≅4840	Very strong	Recessive				Hu et al., 1995
G224D	Exo	As wt	≅270	Slight	Recessive				Hu et al., 1995
H225D	Exo	As wt	≅500	Moderate	Recessive				Hu et al., 1995
D230A	Exo	3.6–5.6%	≅220–250	Moderate				↓↓	Foury and Vanderstraeten, 1992; Vanderstraeten et al., 1998
S304L	Exo	As wt	≅120	Slight	Recessive				Hu et al., 1995
C344G	Exo	As wt	≅10					↓	Foury and Vanderstraeten, 1992
D347A	Exo	7.9%	≅120			↓		↓↓	Foury and Vanderstraeten, 1992
T351I	Exo	As wt	≅220	Slight	Recessive				Hu et al., 1995
T716I	Pol	As wt	≅15	Very strong	Dominant				Hu et al., 1995
E724K	Pol	30%	≅65	Very strong	Dominant				Hu et al., 1995
P851L	Pol	As wt	≅55	Slight	Dominant				Hu et al., 1995
R1001	CTE	20%	≅50	Slight					Hu et al., 1995
Δ351^d^	Pol/CTE	100%							Young et al., 2006
Δ279^d^	Pol/CTE	100%				Absent			Young et al., 2006; Viikov et al., 2012
Δ216^d^	CTE	74–98%	10–100			↓	↑	As wt	Young et al., 2006; Viikov et al., 2012
Δ175^d^	CTE	As wt	As wt			As wt	↑	As wt	Young et al., 2006; Viikov et al., 2012

a *“As wt” means that the petite frequency is not significantly different form that of the wild type strain*.

b *Thermosensitivity regards petite frequency and oxidative growth at 36 or 37°C*.

c *↓ means that the activity/processivity is moderately reduced, ↓↓ means that the activity/processivity is strongly reduced, ↑ means that the processivity is increased*.

d *Deletions of C-terminal regions*.

The 3′–5′ exonuclease domain has a proofreading activity which, in its human counterpart, contributes at least 20-fold to the fidelity of replication (Longley et al., 2001). Specific mutations in *exo1*, *exo2*, and *exo3* drastically reduce the exonuclease activity and consequently, the fidelity of replication (Foury and Vanderstraeten, 1992; Hu et al., 1995; Vanderstraeten et al., 1998) (Table 1). In addition, some of these mutations, such as D171A and the double mutant D171A/D230A, not only decrease the proofreading activity, but also determine an increase of the mismatch extension, with a double negative effect on the replication fidelity. The analysis of these mutants showed that the Mip1 proofreading activity mainly corrects transversions, whereas the protein Msh1, which is involved in the post-replication mismatch repair, mainly corrects transitions. In addition, many mutations in the exo domain, as previously mentioned, determine an increase in the percentage of *petite* colonies in the clonal population. This suggests that the two catalytic domains do not carry out their activities independently of each other and, in particular, that mutations in the exo domain decrease the polymerase activity.

Human DNA polymerase γ consists of a catalytic subunit (POLGA) and two accessory subunits (POLGB) (Yakubovskaya et al., 2006). POLGB is a small subunit which binds the catalytic subunit, binds tightly the DNA and increases the processivity of the whole holoenzyme (Lim et al., 1999). The spacer domain of POLGA is divided into two subdomains: an intrinsic processivity (IP) subdomain and a long accessory-interacting determinant (AID) subdomain (Lee et al., 2009). The first one is responsible for the intrinsic processivity of the catalytic subunit, whereas the second, which interacts with the accessory subunits, is responsible for enhanced processivity. On the contrary, Mip1 is a monomeric protein (Lucas et al., 2004; Viikov et al., 2011). The absence of the accessory subunit explains why the Mip1 spacer is shorter than that of POLGA of higher eukaryotes. Indeed, most of the sequence that corresponds to the AID subdomain is absent in Mip1, and the IP subdomain has a shorter sequence than the human counterpart (Lee et al., 2009). Nevertheless, Mip1 is highly processive: this probably indicates that the mechanisms responsible for processivity in Mip1 and in human Pol γ are partially different (Viikov et al., 2011).

The CTE is a specific region of fungal Pol γ and its length is variable from species to species, reaching its maximum in *Saccharomycetales* (Young et al., 2006). In Mip1, the CTE corresponds to residues 975–1254. Deletion of the last 216 amino acids, among which the most conserved residues are located, leads to a sharp increase of extended mutability (*petite* frequency of 98% after 24 h) and point mutability (10–100-fold increase in Ery^R^ mutants), while the deletion of the last 175 poorly conserved amino acids leads to a wild type phenotype: this indicates that some residues between 1038 and 1079 are critical for the activity and replication fidelity of Mip1 (Young et al., 2006). In addition, arginine 1001, a very conserved residue in fungal polymerases, is important for the polymerase activity, since replacing it with isoleucine causes a *petite* frequency of 20% and a 50-fold higher point mutability compared to the wild type (Hu et al., 1995). *In vitro* analysis showed that the region 1038–1079, despite containing several positively charged residues, is important for efficient polymerization but not for processivity, which is increased in the absence of this region (Viikov et al., 2012). However, the exonuclease activity of the mutant Mip1 lacking the last 216 amino acids is similar to that of wild type Mip1, so that in the presence of low concentrations of dNTPs the balance between the exonuclease activity and the polymerase activity is tipped in favor of the first. Similarly, the strand displacement activity is halved (Viikov et al., 2012).

## Polymorphisms in Mip1

Alignment of Mip1 sequences from several strains obtained by the Sanger *Saccharomyces* genome resequencing project (https://www.sanger.ac.uk/research/projects/genomeinformatics/sgrp.html) and by the *Saccharomyces* genome database (www.yeastgenome.org) reveals the presence of 28 amino acid substitutions (Baruffini et al., 2007a) (Supplementary Table 1). The strains with the greatest differences are the reference strain S288C, which possesses the allele named *MIP1[S]*, and strain Sigma1278b, which possesses the allele *MIP1*[Σ] (Baruffini et al., 2007a). Most of the substitutions are semi-conservative (Supplementary Table 1, green amino acids) or map in positions which are poorly conserved in Pol γ from different fungal and animal organisms (yellow amino acids). Two exceptions are the amino acid 357, which is E in S288C-derived strains and is K in all the other strains, and the amino acid 661, which is A in the former strains but is T in the latter ones. Amino acids E357 and A661 were transmitted to S288C by its ancestor EM93, a heterozygous diploid strain which contains one K357-T661 allele, and one E357-A661 allele. The latter allele is also present in strains BY474X, which are isogenic to S288C and which were used to construct the deletant strain collections, and in the W303-1B strain, which shares a common origin with S288C and is one of the most used strains in the analysis of mitochondrial phenotypes (Schacherer et al., 2007). We and others have shown that the presence of alanine at position 661 results both in higher extended mtDNA mutability (2–2.5% *petite* frequency for strain W303-1B compared to 0.5–1% for the strains with T661) and in increased thermosensitivity (5–20% *petite* frequency at 36°C, 25–40% at 37°C, 60–70% at 38°C for strain W303-1B compared to 0.5–3%) (Baruffini et al., 2007a; Young and Court, 2009). This substitution is then responsible for about one fourth of the *petite* mutability in strains BY474X (Dimitrov et al., 2009). A661 also determines a 3-4-fold increase of mtDNA point mutability (Baruffini et al., 2007a). The amino acid E357 is responsible for a 2-3-fold increase of the mtDNA point mutability, but has no effects on the mtDNA extended mutability.

Due to the presence of amino acid substitutions affecting the mitochondrial mutability, it is critical to choose correctly the genetic background for evaluating the effects of substitutions in Mip1. The use of a strain containing the high mutator allele *MIP1[S]*, with increased basal mitochondrial mutability, allows one to note even small effects consequent to amino acid substitutions, since the gap resulting from the introduction of pathological mutations is larger. On the other hand, the use of a strain containing the low mutator alleles *MIP1*[Σ] or *MIP1[S]*^*A*661*T*^ allows the study of highly deleterious mutations, which in the *MIP1[S]* background could lead to a total loss of mtDNA (Baruffini et al., 2007b).

## Mip1 interactions

Physical or genetic interactions between Mip1 and a few mitochondrial and non-mitochondrial proteins have been reported. Physical interaction with Sed1 was demonstrated by co-immunoprecipitation experiments (Phadnis and Sia, 2004). Sed1 is located both on the cell surface and within the inner mitochondrial membrane (IMM). Deletion of *SED1* leads to a 3.2-fold increase of Ery^R^ mutability and to a 4.3-fold increase of *petite* mutability, and to a decrease of Cox3 protein levels. Furthermore, Mip1 levels are reduced 3-fold. On the basis of these observations, it has been hypothesized that Sed1 could assist other proteins, including Mip1, in the mitochondrial import, leading to a reduction of their levels in its absence. Sed1p could also be associated with the mitochondrial replication machinery (MRM), at the site of mtDNA binding to the inner membrane, leading to a stabilization of the proteins of this complex, such as Mip1 (Phadnis and Sia, 2004). Mip1 is also part of a complex which includes Abf2, Mgm101 and the mtDNA. Thanks to the Mgm101 interaction with transmembrane protein Mmm1 in the outer membrane, this complex takes part to a two-membrane-spanning replisome, which is essential for mtDNA maintenance (Meeusen and Nunnari, 2003). This complex could also include Pif1, the mitochondrial helicase involved in recombination (Cheng and Ivessa, 2010).

At a high temperature (>37°C), the maintenance of mitochondrial genome is partially impaired. It has been demonstrated that at a non-permissive temperature Mip1 is partially misfolded, thus explaining the increase of *petite* frequency. Most Mip1 molecules are misfolded in the strain mutated in *MDJ1*, which encodes a mitochondrial co-chaperone which has a role in maintaining the active conformation of several proteins, including Mip1, at elevated temperatures (Duchniewicz et al., 1999). Moreover, after a heat shock at 46–48°C, Mip1 can partially reactivate its active conformation only in the presence of the cochaperone Mdj1 and the bichaperones Ssc1(Hsp70)-Hsp78 (Germaniuk et al., 2002; Lewandowska et al., 2006).

The first identified genetic interactor of *MIP1* was *RNR1*, which encodes the large subunit of the ribonucleotide reductase, which catalyzes the reduction of rNDPs to dNDPs (Elledge and Davis, 1990; reviewed in Nordlund and Reichard, 2006). Foury and coworkers found that *RNR1* is a multicopy suppressor of the thermosensitive mutation *mip1-1* capable of restoring the growth of the mutant strain at 36°C; moreover, in the diploid strain *MIP1*/*mip1*Δ, which has a *petite* frequency of about 45% at 37°C, overexpression of *RNR1* reduces the extended mtDNA mutability approximately 2-fold (Lecrenier and Foury, 1995). Subsequently, it was found that the overexpression of *RNR1* reduced the detrimental effects on extended mtDNA mutability caused by different pathological mutations modeled in Mip1 (Baruffini et al., 2006; Stumpf et al., 2010). The rescue by *RNR1* overexpression is probably due to the increase of the dNTP pools, which are 3-8-fold higher. The same levels of rescue can be achieved by deletion of *SML1*, which encodes a protein that inhibits the ribonucleotide reductase activity by binding and sequestering Rnr1 (Chabes et al., 1999; Zhao et al., 2000). The deletion of *SML1* had the same effect of *RNR1* overexpression both on the *mip1-1* mutant and on the pathological Mip1 variants (Zhao et al., 1998; Baruffini et al., 2006, 2012).

A third genetic interactor is *MSH1*, which encodes the only mitochondrial protein responsible for the mismatch repair. Deletion or overexpression of *MSH1* affects both the point and the extended mtDNA mutability. In fact, *msh1*Δ cells become *petite* within a few generations (Reenan and Kolodner, 1992), whereas strains overexpressing *MSH1* display an increased *petite* frequency (Dzierzbicki et al., 2004). The deletion of a single *MSH1* copy in a diploid strain increases 7-fold the point mutability (Chi and Kolodner, 1994), whereas a moderate overexpression reduces the Ery^R^ mutability (Koprowski et al., 2002). Deletion of *MSH1* in hyper- or hypomutator *mip1* mutant strains affects the point mutability, as described for the *mip1*^*R*233*W*^ mutant strain, where the effects due to the two mutations were additive (Foury and Szczepanowska, 2011).

The last genetic interactors known so far are *REV3* and *REV7*. These encode the two subunits of Pol ζ, which is involved in the error-prone translesion synthesis (TLS), and *REV1*. *REV1* encodes a deoxycytidyl transferase involved in the repair through TLS of abasic sites and adducted guanines in damaged DNA and forms a complex with Pol ζ. These three proteins have also been detected in mitochondria (Zhang et al., 2006). The deletion of each of these three genes reduced the frequency of spontaneous or UV-induced -1 frameshift mutations in the mtDNA about 5-20-fold, but at the same time it increased 2-30-fold the spontaneous or UV-induced point mutability (Zhang et al., 2006; Kalifa and Sia, 2007). This suggests that mitochondrial TLS system is more error-prone than that of Pol ζ, and may be represented by Mip1 itself (Kalifa and Sia, 2007). Furthermore, the deletion of *REV3*, *REV7*, or *REV1* in a *mip1Δ* strain unable to lose the mtDNA showed how Pol ζ and Pol γ belong to the same epistatic group, while Rev1 belongs to a different group. In contrast, overexpression of *REV3* and *REV1*, as discussed in more detail later, is able to reduce the *petite* mutability due to pathological substitutions in Mip1 (Baruffini et al., 2012).

## Validation of Pol γ pathological mutations in yeast

In humans, mutations in *POLG* cause many mitochondrial pathologies, like PEO, Alpers' syndrome and ataxia-neuropathy syndrome. These are all characterized by instability of mtDNA, i.e., mtDNA depletion and/or large scale deletions, which result in impaired mitochondrial function in several tissues with different degrees of severity (reviewed in Stumpf and Copeland, 2011). From the first observation that a disease associated with multiple deletions of mtDNA was caused by a mutation in a nuclear gene (Zeviani et al., 1989) later identified as *POLG* and from the first identification of mutations in *POLG* as the cause of PEO (Van Goethem et al., 2001), almost 250 disease-associated mutations have been documented. These are compiled at the Human DNA Polymerase γ Mutation Database (http://tools.niehs.nih.gov/polg/), which reports the genetics related to the different mutations and a description of the associated phenotypes. Most of the mutations were identified in compound heterozygosity, i.e., a mutation was present in the maternal allele and a different mutation in the paternal allele. Some patients carry only a single mutation in each allele, but most patients carry two mutations in one or both the alleles. In these cases, it is unclear how the two substitutions in the same allele contribute to the pathological phenotype. In other cases, patients carry both a putative pathological mutation and an amino acid substitution considered as a neutral polymorphism in the same allele, and determining whether the polymorphism can influence the severity of the disease should be of great interest. Moreover, it is often not possible to assess the dominance/recessivity of pathological mutations, especially when the family history is incomplete or absent.

Yeast has been proven to be an ideal genetic system to obtain such information and to validate *in vivo* the pathogenicity of Pol γ mutations, i.e., to establish relations between a mutation and the associated phenotypes. For validation in yeast, the *MIP1* wild type residue is substituted with the corresponding residue found in patients. When the human mutation involves a conserved residue, the corresponding yeast *MIP1* codon can be directly mutated, thus producing the “pathological” allele. When the Pol γ mutated residue is not conserved, but the surrounding stretch is, it is possible to produce a “humanized wild type allele” by replacing the Mip1 amino acid with the wild type amino acid present at the equivalent position of Pol γ. The “humanized wild type allele” is then mutagenized to obtain the “pathological allele.” In order to correctly substitute the orthologs residue, the sequence alignment between human and yeast mtDNA polymerase has to be done unambiguously. However, not all residues involved in diseases are located in conserved regions. Another great advantage of yeast is the possibility of working in the same genetic background, in which the mutant strains differ from each other only for the *mip1* mutant allele. For the validation of disease-related mutations, *mip1* mutant alleles are introduced in a *mip1Δ* strain. Since the deletion of the chromosomal *MIP1* gene makes the cells immediately and irreversibly *rho*^0^, the deletion of *MIP1* has to be carried out in cells containing a plasmid-borne wild type *MIP1* allele, which can be removed by plasmid shuffling only after its replacement with the mutant allele under analysis (as in Baruffini et al., 2006, 2007a,b, 2010, 2011; Spinazzola et al., 2009; Stricker et al., 2009). Another strategy involves first disrupting the *MIP1* wild type gene thus obtaining a *mip1*Δ*rho*^0^ strain, and then introducing the mutant allele at the chromosomal locus or in a plasmid. Functional mtDNA is subsequently reintroduced either by crossing with wild type *rho*^+^ cells and sporulation (as in Stuart et al., 2006; Stumpf et al., 2010) or by the cytoduction techniques (as in Szczepanowska and Foury, 2010). However, it must be underlined that the use of yeast Mip1 to model pathological mutations also has some shortcomings. Amino acids which are not conserved and are not in a conserved stretch cannot be studied. In addition, the validation is based on the general assumption that if an amino acid is conserved between Mip1 and human Pol γ, the substitution of that amino acid in one protein can predict the effect of the mutation in the second protein. However, this assumption is not applicable to all amino acids, especially if the amino acid lies far from the active site or has a second function specific to human POLGA, such as the binding of POLGB, as in the case of the amino acid A467 (Chan et al., 2005a).

The obtained mutant strains are then analyzed to assess the effects of the *mip1* mutation on: (i) oxidative growth, by spot assays on media supplemented with non-fermentative carbon sources; (ii) mtDNA extended mutability, measured as frequency of *petite* mutants; (iii) mtDNA point mutability measured as frequency of Ery^R^ mutants, which arise from specific point mutations in the mitochondrial 21S rRNA encoding gene. These analyses are carried out at the optimal growth temperature, which in yeast is 28–30°C, but also at a higher temperature (37°C), which is more stressful for mitochondrial metabolism, in order to assess thermosensitivity of mutant Mip1.

It is also possible to determine whether the pathological mutation is responsible for mtDNA deletions or complete depletion by analyzing the nature of *petite* mutants produced (*rho*^−^ or *rho^0^*). For this purpose, different techniques have been applied. The most frequently used method is crossing a large number of defined *petite* mutants (tested strains) with several *mit^−^* strains (tester strains) harboring point mutations in mitochondrial genes encoding respiratory proteins. The method is based on the capacity of *rho*^−^ genomes to retrieve *mit^−^* genomes to wild type through homologs recombination. If restoration of respiratory competence in diploids is observed, it means that the *rho*^−^ genome has retained a DNA fragment encompassing the *mit^−^* mutation (as in Baruffini et al., 2006, 2007b, 2011; Szczepanowska and Foury, 2010). This method was also validated by Southern-blot analysis (Baruffini et al., 2006). *Petite* mutants can also be examined by confocal fluorescence microscopy following 40,60-diamidino-2-phenylindole hydrochloride (DAPI) staining of cytoplasm, which in yeast also stains mtDNA (as in Stuart et al., 2006; Qian et al., 2014). In both *rho*^−^ cells and *mit^−^* cells stained with DAPI, several small spots can be observed under the surface of the cells, whereas these spots are absent in *rho*^0^ due to the lack of mtDNA.

To examine the quantity and integrity of the mitochondrial genomes in *petite* mutants, quantitative PCR (qPCR) methods were used (as in Stuart et al., 2006; Qian et al., 2014). The copy number of mitochondrial genomes was determined by qPCR of short mitochondrial targets: no detectable PCR products indicate the absence of mtDNA. The integrity of mtDNAs was determined by qPCR of long mitochondrial targets. The relative amplification of the long mtDNA fragment in *petite* cells, compared to that of the reference wild type controls, provides information about the presence of damaged DNA that blocks the PCR polymerase (as in Stuart et al., 2006). Altogether, these experimental approaches provide relevant information about the molecular mechanism of the replication defect associated with the disease.

Besides these analyses, other biochemical parameters regarding specific Mip1 activity can also be analyzed. For example mtDNA polymerase activity can be analyzed by gap filling experiments, processivity, DNA binding affinity, exonuclease activity, dNTP misincorporation (as in Szczepanowska and Foury, 2010).

In Supplementary Table 2, a list of results concerning validation in yeast of several *POLG* mutations is presented. All the mutations involve amino acids that are conserved between human and yeast mtDNA polymerase or located in a conserved region, except human A467T, which lies in the linker domain. In humans, this mutation was often found in compound with other mutations. However, homozygous subjects were also described, which indicates that a specific pathological defect is associated with this mutation.

Validation results obtained by different authors are in general coherent, except for a few cases, probably due to the different (non-isogenic) background of the strains used, which results in a higher variability and thus in a non-statistically significant difference. Considering only mutations for which a pathological role has been demonstrated or postulated on the basis of several observations, the prediction capability of the yeast model systems fluctuates from 70% (as in Stumpf et al., 2010) to 100% (as in Baruffini et al., 2007b, 2011). An example of discrepancy concerns human mutation R574W (yeast R467W). Two independent laboratories have obtained similar results using different yeast strains, showing that the presence of the yR467W mutation increases both extended and point mtDNA mutability (Szczepanowska and Foury, 2010; Baruffini et al., 2011); on the contrary, on the basis of results reported by Stumpf et al. (2010), the yR467W mutation has to be considered a neutral change in yeast. This discrepancy can be explained by the onset of a second mutation, capable of suppressing the phenotypic effect of the yR467W mutation, or by the use of a genetic background with a high basal *petite* frequency (approximately 10%). Indeed, in our experience, a higher basal *petite* frequency is associated with a higher standard deviation between experiments, so that it is more difficult to demonstrate that a small difference is statistically significant.

One of the advantages offered by yeast is the possibility of obtaining accurate information on the dominance/recessivity of mutations, which is not always easy and straightforward in patients. The dominance/recessivity is determined analyzing mtDNA mutability in the so-called “heteroallelic” strains, in which both a wild type *MIP*1 copy and a *mip1* mutant allele are present, thus mimicking the human diploid condition. For instance, yeast studies have been crucial in establishing the dominance of the extremely severe mutation hE895G (yE698G), found in a child dead 36 h after birth. Due to unclear family history, it could not be established whether the hE895G mutation was inherited as a dominant or recessive trait, as well as whether it was transmitted from the reportedly normal proband father, whose DNA was not available for the study, or was the result of a *de novo* event. The latter hypothesis was supported by the results obtained in the heteroallelic *MIP1/mip1*^*E*698*G*^ yeast strain, which demonstrated the negative dominant character of the hE698G mutation (Spinazzola et al., 2009). The dominant character of hH932Y (yH734Y) and the recessive character of hG1051R (yG807R) were also clearly defined through yeast studies (Baruffini et al., 2007b).

Determination of mtDNA mutability in heteroallelic strains not only allows one to determine the dominance/recessivity of a mutation, but also to understand whether the mutation leads to a gain of function (i.e., dominant negative) or a loss of function (i.e., null allele). In fact an increase of *petite* accumulation was observed in a strain carrying a single copy (hemiallelic strain), compared to a strain carrying two copies of wild type *MIP1* (homoallelic strain), indicating haploinsufficiency (Baruffini et al., 2006). So if the heteroallelic *MIP1/mip1* strain shows a *petite* frequency higher than the homoallelic *MIP1/MIP1* but similar to the hemiallelic *MIP1/mip1Δ*, the mutation determines loss of function and is dominant by haploinsufficiency. On the other hand, if the mutant strain shows a *petite* frequency higher than that of the hemiallelic strain, the mutation is considered dominant negative. It is necessary to underline that, so far, almost all the putative dominant mutations in humans are dominant negative in yeast, whereas mutations that cause a total loss of function in yeast are recessive in humans and often associated with severe diseases.

Yeast is not only suitable for validating mutations and clarifying the pathogenicity of mutations. In some cases, it can also have a “predictive role.” This was the case of mutation hS305R, found in two related subjects. One patient carried the hS305R mutation in compound heterozygous with a second mutation, whereas the second one was heterozygous for the hS305R mutation alone, suggesting that the mutation, which was inherited from the mother, behaves as dominant. In yeast, the corresponding yC261R mutation displayed a recessive phenotype that was then divergent from the clinical feature of the second patient. These data obtained in yeast prompted the authors to look for another mutation. Additional analysis on the patient's DNA was not feasible, but further examination of parents' DNA allowed to detect an additional pathogenic mutation, hP1073L, which was identified in the father and was missed in the first genetic analysis. This confirmed that the second patient was also compound heterozygous for two allelic mutations (Baruffini et al., 2011). Therefore, the discrepancy between the results obtained in yeast and the clinical features may in some case predict the presence of a cryptic, undetected mutation in the patients second allele, as postulated in the case of mutation hR386H (yI334H).

Patients often carry more than one *POLG* mutation, *in cis* or *in trans*, and the specific contribution of each mutation to the disease cannot be established. In yeast, mutations can be studied alone or in combination, so it is possible to clearly evaluate the contribution of each mutation to the pathology. Functional interactions of *mip1* mutations *in cis* were studied by introducing both mutations in the same *mip1* allele, and *in trans* by transforming the *mip1*Δ strain with the two plasmid-borne mutant alleles (Supplementary Table 3). For instance, in the case of mutation hH932Y (yH734Y), found *in trans* with hG1051R (yG807R), the comparison of the severity of the yH734Y and yG807R mutations, alone or in compound, strongly suggested that the major contributor to the disease was hH932Y, even though a strong synergistic effect of the two mutations, when associated, was confirmed (Baruffini et al., 2007b).

A different degree of severity of Alpers' disease has been described in patients carrying the common A467T mutation in compound with mutation G303R, R574W or P625R, suggesting that the phenotypic differences observed are likely to be ascribed at least in part to the presence of these additional mutations. Results in yeast demonstrated that hG303R (yG259R) is highly detrimental, hR574W (yR467W) produces an intermediate phenotype which is also very severe but less damaging than G303R, and hP625R (P513R) induces a mild defect at 28°C but a strong increase of mtDNA mutability at 37°C. The detrimental effects observed in yeast correlated to the severity of this phenotype in humans, thus explaining the phenotypic modulation of the observed clinical features.

In some cases pathological mutations are present in compound with single nucleotide polymorphisms (SNP). This raises the question of whether these substitutions are neutral or can act as phenotypic modifiers which lead to more severe clinical phenotypes. This was the case of the hA889T (yA692T) mutation present in cis-compound with the SNP E1143G (E900G). Comparing the effect of the *mip1*^*A*692*T*^^+^^*E*900*G*^ allele, which carries the two mutations in compound, and the effect of *mip1*^*A*692*T*^ or *mip1*^*E*900*G*^ mutant alleles, demonstrated that the presence of the two substitutions *in cis* exerts a deleterious synergistic effect on the mtDNA mutability, thus indicating that E900G is not a neutral polymorphism if in compound with another mutation (Baruffini et al., 2007b).

## Besides validation: molecular defects associated with Mip1 mutations

Studies on Pol γ mutations in yeast, animal models, cell culture, *in vitro* and *in silico* analysis have clarified some of the molecular defects associated with different mutations.

In yeast it has been observed that the mutations L210P (human L244P), G651S (hG848S), A692T (hA889T), H734Y (hY932Y), G807R (hG1051R), and E900G (hE1143G) lead to a 50 to 90%, reduction in mitochondrial protein levels. Reduced protein levels may account, at least in part, for the increase of *petite* mutability associated with these mutations (Baruffini et al., 2007b; Szczepanowska and Foury, 2010). Since the expression levels of such Mip1 variants are normal the decrease of soluble mitochondrial proteins suggests either that the mutant proteins are not transported properly into the mitochondria or that proteins are misfolded and/or degraded in mitochondria. Although the analyses performed on patients' cells are limited, it was observed that a decrease of Pol γ holoenzyme levels occurs in fibroblasts from patients bearing mutations R232H and G848S *in trans* (75% reduction), and mutations A467T and T914P *in trans* (45% reduction) (Taanman et al., 2008). A reduction in Pol γ catalytic subunit was also observed in a patient bearing the mutation T9214P (Roos et al., 2013) and, in another patient, the complete lack of POLGA containing the stop mutation E873X, due to nonsense-mediated mRNA decay, was reported (Chan et al., 2005b).

Most of the mutations in *POLG* determine alterations of the mtDNA polymerase biochemical properties, i.e., decrease of catalytic activity, processivity, DNA binding affinity, and/or binding affinity for the incoming dNTP (reviewed in Chan and Copeland, 2009; Stumpf and Copeland, 2011). Regarding the Mip1 mutant, the following was observed: (1) decrease in the gap-filling DNA synthesis associated with mutations L260R (hL304R), R265L (hR309L), R265H (hR309H), F268R (hW312R), R467W (hR574W), G651S (hG848S), A692T (hA889T), H734Y (hY932Y), G807R (hG1051R); (2) decrease in processivity and of DNA binding affinity in the case of mutations L360R, R265L, R265H, R467W, and F268R; (3) increase in dNTP misincorporation in the case of mutations L260R and R265H; (4) increase in Exo/Pol ratio in the case of mutations L260R, R265L, R265H, and F268R (Baruffini et al., 2007b; Szczepanowska and Foury, 2010). These data were confirmed for human Pol γ harboring mutation G848S, showing less than 1% of polymerase activity and a 5-fold increase in K_*d*_(DNA) (Kasiviswanathan et al., 2009).

In contrast, the exonuclease activity is only slightly or not at all reduced, even in the case of mutations in the exo domain, suggesting that the defects of mtDNA replication are not due to defects of proofreading activity but to deficiency of polymerase activity (Szczepanowska and Foury, 2010). In yeast, the absence of the Mip1 exonuclease activity results in a 160-fold increase of the frequency of deletions between 21 bp direct repeats (Phadnis et al., 2005; Stumpf and Copeland, 2013). Seven mutations in the exo domain did not cause an increase of this type of deletions, further indicating that the exonuclease activity is scarcely or not at all affected (Stumpf and Copeland, 2013).

Regarding the dominant negative mutations, two main hypotheses have been proposed to explain this dominance. Some mutations completely inhibit the polymerase activity of Pol γ, but not the DNA binding affinity. Thus, the mutant polymerase that binds the DNA with similar affinity to that of wild type Pol γ may block the replication but may also prevent the binding of the wild type enzyme. For example, the Y955C mutation, which is dominant in both yeast and humans, prevents the polymerase from synthesizing DNA, especially in the case of the incorporation of dATP:T, but it does not change the K_*d*_(DNA) (Graziewicz et al., 2004; Estep and Johnson, 2011). On the other hand, some mutant Pol γ variants may directly cause lesions to DNA. In the diploid strain containing a wild type *MIP1* allele and a *mip1* allele harboring mutation Y757C, which corresponds to human Y955C, an accumulation of mtDNA lesions was found (Stuart et al., 2006). In addition, in mice expressing a cardiac targeted Y955C variant, an accumulation of the oxidized nucleotide 8-hydroxy-2-deoxyguanosine (8-OHdG) in the mtDNA (Lewis et al., 2007) was observed, probably because the Y955C Pol γ displays a reduced discrimination for incorporation of 8-oxo-dGTP or for translesion synthesis opposite to 8-oxo-dG (Graziewicz et al., 2007). However, more recently it was shown that physiological effects of the Y955C mutation might not be due to an increased incorporation of 8-oxo-dG, since the incorporation rate of 8-oxo-dG is low in wt Pol γ and further decreased 500-fold in Y955C Pol γ (Hanes and Johnson, 2007; Estep and Johnson, 2011).

On the basis of information obtained from several studies, including studies in yeast and an *in silico* analysis, the majority of mutations in *POLG* were included in five clusters, depending on the defect associated with the mtDNA polymerase (Euro et al., 2011; Farnum et al., 2014): cluster 1, mutations in the polymerase domain, which are mostly dominant and which affect the polymerase activity; cluster 2, recessive mutations in the spacer domain which affect the upstream DNA-binding channel; cluster 3, recessive mutations in the exo domain and in the fingers subdomain which are associated with a Pol γ specific functional module involved in the partitioning of the DNA substrate between the exo and the pol catalytic sites; cluster 4, recessive mutations in the exo domain which influence the bond with one of the accessory subunit; cluster 5, recessive mutations of the IP subdomain which affect the binding to other proteins of the replisome.

## Besides validation: chemical and genetic rescue of the phenotype

By using yeast, it is possible to easily identify, in short times, molecules or genes able to reduce the effects of pathological substitutions.

Regarding the chemical rescue, active molecules were identified by analyzing their ability to restore oxidative growth and/or to reduce *petite* frequency of *mip1* mutants grown in their presence. The identification of these molecules has a double significance. Firstly, on the basis of the mechanisms by which the drugs act, it is possible to deduce information on the molecular defects caused by the Mip1 mutation. Secondly, identification of such drugs can pave the way for studies on their therapeutic potential. It has been demonstrated in yeast that the administration of lipoic acid or MitoQ, two mitochondrial antioxidant molecules, reduces the *petite* mutability due to some *mip1* mutations (Baruffini et al., 2006, 2011, 2012). Out of all the studied mutations, the dominant mutations in the polymerase domain, in particular the mutation Y757C (hY955C), are particularly sensitive. This observation is consistent with the fact that, as reported in the previous section, human Pol γ harboring Y955C leads to increased incorporation of 8-OHdG. Thus, it is possible that supplementation with antioxidants reduces the levels of oxidized bases, which would consequently be incorporated into the mtDNA.

Regarding the genetic rescue, both the overexpression of *RNR1* and the deletion of *SML1* reduce the *petite* mutability in the ts mutant *mip1-1* by increasing the concentration of dNTP. This was also observed for most of the pathological mutations introduced in *MIP1* (Baruffini et al., 2006, 2011, 2012; Stumpf et al., 2010), although at a different extent. It is reasonable to think that the rescue is greater when the ability of the Mip1 mutant to bind the incoming dNTP is more compromised. This was demonstrated for the H734Y mutation, for which *RNR1* overexpression causes a 5-fold decrease of *petite* frequency. *In vitro* analysis on human Pol γ containing the corresponding H932Y mutation showed that the affinity for the incoming dNTP is reduced approximately 200-fold compared to the wild type (Stumpf et al., 2010). The increase of the dNTP pools as a rescue mechanism was also demonstrated by the observation that the processivity defect of human DNA polymerase γ harboring the Y955C mutation was overcome by increasing the dATP or dTTP concentration during *in vitro* synthesis of mtDNA fragments (Atanassova et al., 2011). Supplementation of dNTPs precursors as a potential therapeutic agent was shown on differentiating myotubes of a patient harboring mutations in *POLG*, in which the addition of dAMP and dGMP slightly increased the levels of mtDNA (Bulst et al., 2009). In addition, supplementing myotubes of patients harboring mutations in *POLG* with specific combinations of the four dNMPs showed near normalization of the mtDNA levels (Bulst et al., 2012). However, it is necessary to emphasize that the alteration of the dNTPs concentration may also have deleterious consequences (Xu et al., 2008).

Finally, the extended and point mtDNA mutability due to Mip1 mutations is rescued by overexpression of Pol ζ and Rev1 that, in yeast, are also located in the mitochondria (Zhang et al., 2006; Baruffini et al., 2012). Intriguingly, the Mip1 mutations which are rescued by Pol ζ overexpression are not recovered by antioxidant treatment, and vice versa, suggesting that the rescue is exerted through two alternative mechanisms. The first one is most likely related to the ability of Pol ζ to replace Mip1 containing mutations which mainly reduce the catalytic activity. The second one is likely linked to the ability of antioxidant molecules to reduce the concentration of oxidized bases which can be incorporated by Mip1 bearing specific mutations such as Y757C.

## Beyond validation: study of correlation between mutations/polymorphisms in Pol γ and mitochondrial toxicity caused by sodium valproate, NRTIs and environmental mutagens

Sodium valproate (valproic acid, or VPA) is a drug used as an anticonvulsant, and for migraine, bipolar disorder and chronic headache. However, the administration of VPA in some individuals can lead to a fulminant liver failure. The frequency of this adverse effect is very high in patients with Alpers' syndrome bearing mutations in *POLG*, and also in subjects not affected by Alpers' syndrome but carrying Pol γ polymorphisms. Through a combined approach performed on cells of subjects showing episodes of VPA-induced liver toxicity and harboring polymorphisms in *POLG*, and on yeast cells bearing the corresponding *mip1* mutations, it was demonstrated that patients heterozygous for polymorphisms E1143G and Q1236H have a higher probability of developing VPA-induced toxicity (Stewart et al., 2010).

Nucleoside reverse transcriptase inhibitors (NRTIs) are used in the highly active antiretroviral therapy (HAART), which has significantly increased the life expectancy of HIV patients. However, in some patients a prolonged treatment induces side effects, most of which are due to interference of the NRTIs or of their triphosphorylated form with the mitochondrial function. NRTIs are nucleosides analogs in which the hydroxyl moiety in 3′ was substituted with a group that blocks the formation of the 3′-5′ bond in the nascent strand. Several observations suggest that mitochondrial toxicity depends on NRTIs interference with Pol γ activity, resulting in decreased mtDNA levels, especially in the case of pyrimidine analogs, such as stavudine (2′,3′-didehydro-2′,3′-dideoxythymidine, or d4T) and zalcitabine (2′,3′-dideoxycytidine, or ddC) (reviewed in Lee et al., 2003 and Koczor and Lewis, 2010). In addition, some triphosphorylated NRTIs can inhibit the activity of human Pol γ *in vitro* (Johnson et al., 2001; Lim and Copeland, 2001). It has also been demonstrated by *in vitro* experiments that substitutions in Pol γ may alter the mtDNA polymerase discrimination between the correct dNTP and the corresponding dNRTI-TP. This results either in a decreased ability to incorporate the dNTPs, for example in the case of E895G, Y951F, and Y955F substitutions (Lim et al., 2003), or in an increased ability to incorporate the dNRTI-TP, such as in the case of mutation R964C, which causes a 33% decrease of dTTP incorporation efficiency and a 3-fold lower d4TTP discrimination compared to wild type Pol γ (Bailey et al., 2009). Mitochondrial toxicity was observed in a patient homozygous for the R964C mutation after treatment with D4T (Yamanaka et al., 2007), and a correlation between treatment with D4T and mitochondrial toxicity was proposed for subjects heterozygous for the widespread SNP E1143G (Chiappini et al., 2009). We have previously constructed and validated two yeast models useful for studying the correlation between mutations or polymorphisms in *POLG* and mtDNA depletion after treatment with D4T or ddC, (Baruffini and Lodi, 2010; Baruffini et al., 2015). These models were constructed by cloning and introducing in yeast the human *ENT1* gene, which encodes a membrane nucleoside transporter, and the herpes simplex virus *TK1* gene, which encodes a thymidine kinase, or the human *DCK* gene, which encodes a deoxycytidine kinase, in order to allow the proper transport and phosphorylation of the analog. Our results show that, besides R964C and E1143G, other polymorphisms in *POLG* might cause mtDNA instability and depletion following treatment with these NRTIs. However, further studies are needed in human cells.

Alkylating agents, such as methyl methanesulfonate (MMS), also increase mtDNA point mutability besides nuclear DNA mutability. It was shown that administration of MMS in strains harboring mutator *mip1* heterozygous mutations corresponding to human pathological ones further increased the Ery^R^ point mutability due to CG transversions (Stumpf and Copeland, 2014). MMS induced mutability in *mip1* mutant strains seems to be due to an active mechanism, in which the mutant polymerase binds to the DNA and stalls mtDNA replication, resulting in ssmtDNA which can be alkylated by MMS. Trace amounts of CdCl_2_ are also associated with an increase in the *petite* frequency and a decrease in the mtDNA content both in homozygous and *MIP1*/*mip1* heterozygous strains (Stumpf and Copeland, 2014).

## Recent findings and future directions in modeling human mutations and drug discovery

In recent years, research on *MIP1* focused on genetic and molecular suppressors able to reduce point and extended mtDNA mutability due to mtDNA polymerase dysfunctions. By random mutagenesis, a *MIP1* mutation, A256T, was recently identified. This behaves like an antimutator, decreasing 2.2-fold the frequency of Ery^R^ mutants (Foury and Szczepanowska, 2011). The A256 residue is conserved in humans (A300) and is part of a conserved region, suggesting that Pol γ A300T can behave as an antimutator allele. Further experiments in animal models should be performed to prove this hypothesis.

In 2014, Qian and coauthors constructed a “humanized” yeast model in which yeast Mip1 was replaced by human Pol γ. They deleted most of the endogenous *MIP1* gene and cloned both *POLG(1)* and *POLG2* under the *MIP1* promoter and in frame with the *MIP1* MLS region. The *mip1*Δ “humanized” strain, containing both POLGA and POLGB, was able to grow on oxidative carbon sources at a similar rate to *MIP1* wild type strain, indicating that the human holoenzyme can complement the loss of Mip1 and can replicate yeast mtDNA. The complementation was partial, since in the humanized strain the mtDNA levels were reduced to 50% compared to the wild type strain, and both *petite* and Ery^R^ frequencies were doubled. Nevertheless, this system has proven useful to model human Pol γ mutations in yeast. Four mutations were introduced in POLGA (S305R, H932Y, Y951N, and Y955C). The behavior of mutant POLGA strains is very similar to strains harboring the corresponding mutations in *MIP1* (Table 2). In both systems, S305R strongly increases the *petite* frequency and is recessive, whereas H932Y, Y951N, and Y955C make the strain *rho*^0^ (and thus unable to grow on oxidative carbon sources) and are dominant. In both systems, Ery^R^ mutant frequency, which is measured in the diploid strain, increases when each mutation is present in heterozygosis, though at different extents (Qian et al., 2014). Overall, the construction of this “humanized” strain indicates that *MIP1*, used until now in validation studies, is a good model to study the phenotypic consequences of mutations, at least for the conserved amino acids. Furthermore, it provides a novel and invaluable tool to assess the physiological effects of disease-associated mutations directly in human Pol γ itself, thus also allowing to overcome the weaknesses associated to the use of Mip1 discussed above.

**Table 2:** **Comparison between four mutations studied in yeast *mip1* strain and in humanized yeast *POLG* strain**.

**Mutation**	**Yeast *mip1* strain**				**Humanized *POLG* strain**			
	**Haploid strain**		**Diploid strain**		**Haploid strain**		**Diploid strain**	
	**Petite^a^**	**Ery^R^^b^**	**Petite^a^**	**Ery^R^^b^**	**Petite^a^ %**	**Ery^R^^b^**	**Petite^a^**	**Ery^R^^b^**
S305R	84	8	2.1	2	100	ND	2.5	7
H932Y	>99%	10	5–9	10	100	ND	11	58
Y951N^c^	100%	ND	15–25	6	100	ND	15	9
Y955C	100%	ND	35–91	11	100	ND	22	24

*The values are calculated starting from the data found in Baruffini et al. (2006), Stuart et al. (2006); Stumpf et al. (2010) and Qian et al. (2014)*.

a *Petite indicates the petite fold increase compared to the wild type, except in the case of strains for which the petite frequency is >99%*.

b *Ery ^R^ indicates Ery ^R^ fold increase compared to the wild type*.

c Baruffini, unpublished results

*ND, not determinable*.

Recently, yeast has proven to be an excellent tool for drug discovery, in particular in the case of mitochondrial disorders. This is of paramount importance since no established treatment for these pathologies is available so far. Molecules acting as potential therapeutics in disorders associated with primary deficiencies in the mitochondrial ATP synthase were successfully found in yeast by high throughput approaches aimed at identifying chemical suppressors of pathological phenotypes (Couplan et al., 2011; Aiyar et al., 2014). In such a screen, the *fmc1* null mutant, unable to assemble the F1 sector of ATP synthase at high temperatures, was used as yeast model of these pathologies. Taking advantage of the RD thermosensitive phenotype of this mutant, a yeast-based assay was developed in which thousands of chemical compounds from several chemical libraries were tested for their ability to suppress the respiratory growth defect at 37°C. By such a screen, drugs effective on a fmc1 yeast mutant were successfully found. These drugs were also active on strains mutated in *ATP6*, which, if mutated in humans, caused neuropathy, ataxia, and retinitis pigmentosa (NARP) syndrome. Moreover, they have proven to be effective in human cybrids derived from NARP patients, thus validating the yeast-based approach and demonstrating that yeast can be used as a pharmacological model for the study of mitochondrial diseases.

We used a similar high throughput approach, in collaboration with A. Delahodde (I2BC, Université Paris-Sud), to find drugs that could potentially be used in POLG disease therapy. About 1600 molecules included in two chemical libraries were assayed for their ability to restore the oxidative growth of thermosensitive *mip1* strains harboring mutations equivalent to the human pathological ones. Six rescuing molecules were identified, three of which also decreased the frequency of cells depleted of mtDNA at 28°C. The rescue has also been proven effective in a *C. elegans POLG* model. Studies on fibroblasts from patients bearing mutations in *POLG* and experiments aimed at gaining more insight into the molecular mechanism behind the rescue are in progress.

### Conflict of interest statement

The authors declare that the research was conducted in the absence of any commercial or financial relationships that could be construed as a potential conflict of interest.
