# Supplementary material for: DNA polymerase γ and disease: what we have learned from yeast
Source: Front Genet. 2015 Mar 17;6:106. doi: 10.3389/fgene.2015.00106 (PMC4362329; doi:10.3389/fgene.2015.00106)
Supplement: Supplementary file 1 [file Table1.DOCX]

**Supplementary Table 1. Sequence substitutions in Mip1 from different *S. cerevisiae* strains.**

| S8F | F170L | S498F | F576Y | A761T | E1134K |
| --- | --- | --- | --- | --- | --- |
| T36A | I222V | T540M | V614I | N972I | T1176A |
| G50D | I289T | H541N | S616N | S978P | I1188V |
| C61S | **E357K** | L543Q | S631L | N986S |  |
| V149I | K479R | N573S | **A661T** | S991N |  |

First amino acids refer to S288C strain. In green semiconservative substitutions, in yellow substitutions in amino acids which are poorly conserved in eukaryotic Pol γ, in red substitutions which affect mtDNA mutability.

**Supplementary Table 2. Validation of human *POLG* mutations in yeast.**

| Human  mutation | Domain | D/R in human^a^ | Yeast mutation | Phenotypes in yeast | | | | | | | |
| --- | --- | --- | --- | --- | --- | --- | --- | --- | --- | --- | --- |
|  |  |  |  | Fold increase of *petite* frequency^b^ | | Fold increase of Ery^R^ frequency^c^ | | Thermo-sensitivity^d^ | D/R in yeast^e^ | Fold decrease copy number in haploid strain^f^ | References^g^ |
|  |  |  |  | haploid | heteroallelic | haploid | heteroallelic |  |  |  |  |
| H110Y | exo | R? | H84Y | As wt | As wt | As wt | As wt |  |  |  | Stumpf et al., 2010 |
| L244P | exo | R? | L210P | As wt  9.2 | 2.5 | 16 - 84 | 6.3 | Yes | D (haplo) |  | Stumpf et al., 2010  Szczepanowska and Foury 2010 |
| G303R | exo | R? | G259R | 100% | 2.5 | - | 2 |  | D (haplo) | Rho0 | Baruffini et al., 2011 |
| L304R | exo | R | L260R | 100%  4.1  71.3 | 6.3 | As wt  51 | As wt | No | D | Rho0  >210 | Stuart et al., 2006  Stumpf et al., 2010  Szczepanowska and Foury 2010 |
| S305R | exo | R? | C261R | 38.4 | 2.6 | 1.9 | As wt | Yes | D (haplo) |  | Baruffini et al., 2011 |
| Q308H | exo | R? | Q264H | 100% | 5.5 | - | 7.6 |  | D (haplo)? | Rho0 | Stumpf et al., 2010 |
| R309H | exo | R? | R265H | As wt 56.2 | As wt | As wt 58 | As wt | No | D (haplo) |  | Stumpf et al., 2010  Szczepanowska and Foury 2010 |
| R309L | exo | R | R265L | 3.6  27 | 3 | 0.2  4.5 | As wt | No | D (haplo) | 2.7 | Stumpf et al., 2010  Szczepanowska and Foury 2010 |
| W312R | exo | R? | F268W | 71.3 |  | 85 |  | No |  |  | Szczepanowska and Foury 2010 |
| R386H | exo | ? | I334H | 1.5 | As wt | 1.4 | As wt | Yes | R |  | Baruffini et al., 2011 |
| A467T | Linker | R | I416T | As wt | As wt |  |  |  |  | As wt | Stuart et al., 2006 |
| R574W | Linker | R | R467W | 24.8  As wt 23.9 | As wt  As wt | 3.8  As wt 18 | As wt  As wt | Yes | R |  | Baruffini et al., 2011  Stumpf et al., 2010  Szczepanowska and Foury 2010 |
| P625R | Linker | R? | P513R | 1.7 | As wt | As wt |  | Yes | R |  | Baruffini et al., 2011 |
| R807C | pol | R? | R607C | 100% | 3.6 | - | 2.6 |  | D (haplo)? | 105 | Stumpf et al., 2010 |
| R807P | pol | R? | R607P | 7 | 4 | 13 | As wt |  | D (haplo)? | As wt | Stumpf et al., 2010 |
| G848S | pol | R | G651S | 100% 100% | 2.2  2.0 | - | As wt  As wt |  | D (haplo)  D (haplo) | 3.6 | Baruffini et al., 2007  Stumpf et al., 2010 |
| T851A | pol | D? | T654A | 100% | 60 | - | 22 |  | D | 70 | Stumpf et al., 2010 |
| R852C | pol | R? | R655C | As wt | As wt | 0.2 | As wt |  |  |  | Stumpf et al., 2010 |
| R853Q | pol | R? | R656Q | 100% | 88 | - | 30 |  | D |  | Stumpf et al., 2010 |
| R853W | pol | R? | R656W | 100% | 12 | - | 7.3 |  | D |  | Stumpf et al., 2010 |
| A862T | pol | R? | A665T | 100% |  | - |  |  | R |  | Stricker et al., 2009 |
| N864S | pol | R? | N667S | 100% | As wt |  | As wt |  | R | 6.8 | Stumpf et al., 2010 |
| G888S | pol | R? | G691S | 1.9 | As wt | As wt | 2.7 |  | R |  | Stumpf et al., 2010 |
| A889T | pol | D? | A692T | 29.7  1.5 | 3.2  As wt | 3.7  As wt | As wt  As wt | Yes | Weakly D  D (haplo) |  | Baruffini et al., 2007  Stumpf et al., 2010 |
| E895G | pol | D? | E698G | 100% | 12,9 | - |  |  | D |  | Spinazzola et al., 2008 |
| G923D | pol | D? | G725D | 100% | 8.4 | - |  |  | D | Rho0 | Stuart et al., 2006 |
| D930N | pol | R? | D732N | 100% | 5.8 | - | 2.7 |  | D |  | Baruffini et al., 2011 |
| H932Y | pol | ?  ? | H734Y | >99%  100% | 9.2  49 | - | As wt  10 | Yes | D  D |  | Baruffini et al., 2007  Stumpf et al., 2010 |
| R943H | pol | D | R745H | 100% | 40.7 | - |  |  | D | Rho0 | Stuart et al., 2006 |
| K947R | pol | ? | K749R | 100% | 23.7 | - | 5.7 |  | D |  | Baruffini et al., 2011 |
| R953C | pol | D | R755C | As wt | As wt | As wt | As wt |  |  |  | Stumpf et al., 2010 |
| Y955C | pol | D | Y757C | 100%  100% | 21.3  90.9 | - | 11.4 |  | D  D | Rho0 | Baruffini et al.,2006  Stuart et al., 2006 |
| A957P | pol | R? | A759P | 100% | 24 | - | 12 |  | D |  | Stumpf et al., 2010 |
| A957S | pol | D | A759S | 5.3  As wt | As wt  2.3 | 0.2 | As wt |  | D (haplo)? | As wt | Stuart et al., 2006  Stumpf et al., 2010 |
| R964C | pol | R | Q766C | 1,7 | As wt | 2,4 | As wt |  | R |  | Stricker et al., 2009 |
| G1051R | pol | R | G807R | 9.4  As wt | 2  As wt | 10.2  As wt | As wt  As wt | Yes | R |  | Baruffini et al., 2007  Stumpf et al., 2010 |
| P1073L | pol | R? | P829L | 29.0 | 2.2 | 13.4 | 3.3 | Yes | D (haplo) |  | Baruffini et al., 2011 |
| G1076V | pol | ? | G832V | 100% | As wt | - | As wt |  | R | Rho0 | Stumpf et al., 2010 |
| R1096C | pol | R | R853C | 100% | 4.9 | - | 3.9 |  | D (haplo)? |  | Stumpf et al., 2010 |
| R1096H | pol | R? | R853H | 100% | 4.3 | - | 11 |  | D (haplo)? | Rho0 | Stumpf et al., 2010 |
| S1104C | pol | ? | S861C | 100% | As wt | - | 9.4 |  | R | Rho0? | Stumpf et al., 2010 |
| V1106I | pol | R | V863I | 100% | 5.9 | - | 4.3 |  | D (haplo)? | Rho0? | Stumpf et al., 2010 |
| E1143G | pol | R/SNP | E900G | 2.2 | As wt | 1.9 | As wt | Yes | R |  | Baruffini et al., 2007 |
| M1163R | pol | R? | M920R | As wt | As wt | As wt | As wt |  |  |  | Stumpf et al., 2010 |
| F1164I | pol | R | F921I | As wt | As wt | As wt | As wt |  |  |  | Stumpf et al., 2010 |
| D1184N | pol | R | D941N | 3.4 | 1.9 | 3.5 | 1.2 |  | D (haplo)? | >210 | Stumpf et al., 2010 |
| K1191N | pol | R? | K948N | As wt | As wt | As wt | As wt |  |  |  | Stumpf et al., 2010 |
| A889T  +  E1143G | pol + pol |  | A692T  +  E900G | 35.8 | 3.1 | 3.4 |  | Yes | D (neg) |  | Baruffini et al., 2007 |

^a^ “D/R in human” means dominance (D) or recessivity (R). “D?” and “R?” mean that the mutation is likely dominant or recessive, respectively. “?” means that the status of the mutation is ambiguous.

^b^ “As wt” means that the *petite* frequency is not significantly different from that of the wild type strain. If the *petite* frequency is higher than 99% or 100%, these percentages are reported. 100% means that the mutant strain is respiratory deficient.

^c^ “As wt” means that the Ery^R^ frequency is not significantly different from that of the wild type strain.

^d^ Thermosensitivity regards *petite* frequency and oxidative growth at 36°C or 37°C.

^e^ In yeast the mutation is recessive (R) if the heteroallelic strain behaves like the homoallelic strain, it is dominant by haploinsufficiency (D (haplo)) if the heteroallelic strain behaves like the hemiallelic strain and it is negative dominant (D (neg)) if the heteroallelic strain has a *petite* frequency higher than that of the hemiallelic strain.

^f^ The fold decrease of mtDNA content was measured through qPCR.

^g^ The different colors designate different papers in which the mutation was described and the corresponding results obtained.

**Supplementary Table 3. Validation of human compound heterozygous *POLG* mutations in yeast.**

| Human compound heterozygous mutations | Yeast  mutations | Fold increase of *petite* frequency | Fold increase of Ery^R^ frequency | References |
| --- | --- | --- | --- | --- |
| G848S/E1143G | G651S/E900G | 8.6 | - | Baruffini et al., 2007 |
| H932Y/H932Y | H734Y/H734Y | 97.8 | - | Baruffini et al., 2007 |
| G1051R/G1051R | G807R/G807R | 8.7 | 22.1 | Baruffini et al., 2007 |
| H932Y/G1051R | H734Y/G807R | 70.7 | 18.7 | Baruffini et al., 2007 |
| A862T/R964C | A665T/Q766C | 5.2 | 11.3 | Stricker et al., 2009 |
| S305R/P1073L | C261R/P829L | 65.1 | 28.8 | Baruffini et al., 2011 |
| W312R/R574W | F268R/R467W | 5.6 | 18 | Szczepanowska and Foury 2010 |

Colors have the same meaning as in Supplementary Table 2.
